# Preparation of a novel monoclonal antibody against caprine interleukin-17A and its applications in immunofluorescence and immunohistochemistry assays

**DOI:** 10.1186/s12896-019-0543-5

**Published:** 2019-07-17

**Authors:** Yang Gao, Feng Feng Sang, De Lan Meng, Yi Wang, Wen Tao Ma, De Kun Chen

**Affiliations:** College of Veterinary Medicine, Northwest A&F University, Yangling, Shaanxi Province 712100 People’s Republic of China

**Keywords:** Caprine interleukin-17A, Prokaryotic expression, Monoclonal antibody, Immunofluorescence, Immunohistochemistry

## Abstract

**Background:**

Interleukin-17 (IL-17), the characteristic cytokine secreted by T helper 17 lymphocytes (Th17 cells), plays a pivotal role in host defense and many inflammatory or autoimmune diseases. The aim of this study was to obtain purified protein caprine IL-17A (cIL-17A) as an antigen for preparing an IL-17A-specific monoclonal antibody (mAb).

**Results:**

The coding sequence (CDS) region of cIL-17A was cloned from the peripheral blood mononuclear cells (PBMCs) of dairy goats and then inserted into the expression vector PET 32a and transformed into competent TransB (DE3) cells. Recombinant fusion protein obtained under optimized conditions was used to immunize BALB/c mice for preparing monoclonal antibodies. Finally, the supernatants of two hybridoma cell lines showing positive reaction with the recombinant fusion protein and negative reaction with fusion tags of PET 32a were collected for western blot, immunofluorescence (IF) and immunohistochemistry (IHC) analysis. Our results showed that the maximum amount of soluble protein could be obtained directly in the supernatant when the recombinant expression cells were induced by isopropyl-β-d-thiogalactoside (IPTG) at a concentration of 0.3 mmol/L at 16 °C for 42 h. Western blot analysis showed that the mAb H8 could recognize the eukaryotically expressed cIL-17A in the supernatant of transfected HEK293T cells. Immunofluorescence and immunohistochemistry assays showed that mAb H8 could strongly recognize both the eukaryotically expressed and natural cIL-17A.

**Conclusions:**

The monoclonal antibody mAb H8 prepared in this study may be a potential tool for the detection of cIL-17A and beneficial for investigating the pathogenesis of various IL-17-associated diseases.

**Electronic supplementary material:**

The online version of this article (10.1186/s12896-019-0543-5) contains supplementary material, which is available to authorized users.

## Background

Interleukin-17 (IL-17), which was first named cytotoxic T-lymphocyte-associated antigen 8 (CTLA-8) [1], is a type of pro-inflammatory cytokine that is mainly produced by T lymphocytes [2]. The IL-17 family consists of six members: IL-17A, IL-17B, IL-17C, IL-17D, IL-17E, and IL-17F. The commonly denoted “IL-17” refers to IL-17A, while IL-17E is also named IL-25 [2–4]. There are various types of IL-17-secreting cells, including T helper 17 lymphocytes (Th17 cells) [2], IL-17-secreting CD8^+^ cytotoxic T lymphocytes (Tc17 cells) [5, 6], γδ TCR^+^ T lymphocytes (γδ T cells) [7, 8], natural killer T cells (NKT cells) [9, 10], and two subsets of innate lymphoid cells (ILCs), i.e., lymphoid tissue–inducer cells (LTi cells) and RORγt^+^ NCR^−^ ILCs [11–13]. The receptors for IL-17 are widely distributed on various types of tissue cells, especially on epithelial cells and immune cells. The IL-17 receptor family includes IL-17RA, IL-17RB, IL-17RC, IL-17RD and IL-17RE, among which, IL-17RA is a common subunit, and each of the remaining subunits can form a heterodimer with IL-17RA. The receptor IL-17RA/RC can be recognized by the homodimers of IL-17A or IL-17F and by the heterodimer formed by IL-17 and IL-17F [14–16].

IL-17 has been widely investigated in human medicine during the last decade. The biological functions of IL-17 are complex; thus, this cytokine is considered a double-edged sword [17]. On one hand, IL-17 plays a critical role in host defenses against extracellular bacterial and fungal infections [18–21]. On the other hand, it is also involved in the development of many disorders, including autoimmune diseases, inflammation, allergic diseases, and tumor progression [22–26].

In recent years, there have been many studies about IL-17 in the area of veterinary medicine and animal science. It was observed that an increase in the IL-17 mRNA level was associated with neutrophil accumulation during airway inflammation in horses [27], and elevated IL-17 was also detected at mRNA level in another study on the vaccination of chickens with salmonella pathogenicity island [28]. In a DSS-induced colitis model of pigs, the expression of IL-17 was higher in mesenteric lymph nodes than in negative controls, while down-regulation of IL-17 was observed in the duodenum of dogs with inflammatory bowel disease [29, 30]. Mastitis is a common disease in dairy ruminants and often results in great economic losses due to decreased milk production and quality. It has been demonstrated that IL-17 may play an important role in dairy ruminant mastitis [31–33].

While IL-17 is involved in a variety of animal diseases, the lack of species-specific IL-17 monoclonal antibodies greatly hampers the study of these diseases. Thus, it will be very valuable to develop IL-17 monoclonal antibodies to thoroughly elucidate the pathogenesis of IL-17-related animal diseases.

In the present study, the coding sequence (CDS) region of cIL-17A (signal peptide sequence removed) was inserted into the expression vector PET 32a and transformed into the host cell TransB (DE3). After optimization by reducing the inducing temperature and prolonging the inducing time, the soluble recombinant fusion protein cIL-17A was obtained directly in the supernatant. After immunization of BALB/c mice, we obtained two hybridoma clones that secreted monoclonal antibodies that recognized the recombinant fusion protein but not the PET32a fusion tags. Finally one of the hybridoma clones named mAb H8 could recognize the eukaryotically expressed cIL-17A in the supernatants of transfected HEK293T cells. Immunohistochemistry and immunofluorescence assays showed that mAb H8 could strongly recognize the eukaryotically expressed cIL-17A in the transfected HEK293T cells and natural cIL-17A located in mammary gland of dairy goat suffering from clinical mastitis caused by *Staphylococcus aureus*. Thus, the monoclonal antibody mAb H8 prepared in this study was suitable for western blot, immunofluorescence and immunohistochemistry assays.

## Results

### Expression and purification of the recombinant fusion protein cIL-17A

To obtain soluble recombinant fusion protein, the identified *E. coli* TransB (DE3) containing the recombinant expression plasmid cIL-17-PET 32a (detailed in Supplementary Material), which was named “cIL-17-PET 32a-TransB (DE3)”, was induced by various concentrations of IPTG at different temperatures. A series of IPTG concentrations (0.01, 0.05, 0.1, 0.3, 0.5, 0.7 and 1.0 mmol/L) and temperatures (37, 33, 30, 28, 25, 23, 20, 18 and 16 °C) were chosen to optimize the expression conditions. Finally we found that the best yield of the fusion protein could be obtained when cIL-17-PET 32a-TransB (DE3) was induced by 0.3 mmol/L IPTG at 16 °C for 42 h (Additional file 1: Figure S4).

**Fig. 1:**
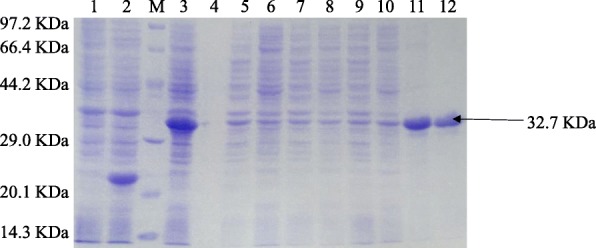
Enlarged cultivation and purification of the recombinant cIL-17A induced at 16 °C for 42 h. M, Premixed protein marker (low); Lane 1, the uninduced cIL-17-PET 32a-TransB (DE3); Lane 2, induced PET 32a-TransB (DE3) with IPTG at a concentration of 0.5 mmol/L; Lane 3, unpurified recombinant cIL-17A; Lane 5–7, eluents after the unpurified supernatant flowed through the column; Lane 8–10, eluents of unrelated proteins after the column was washed with imidazole at a concentrations of 80 mmol/L; Lane 11 and 12, purified recombinant cIL-17A. The position of the recombinant fusion protein is indicated by an arrow

A larger culture of 100 ml of induced cIL-17-PET 32a-TransB (DE3) with IPTG at a concentration of 0.3 mmol/L at 16 °C for 42 h was prepared. After sonification and centrifugation the protein was purified from the supernatant fraction according to the optimized conditions (shown in Supplementary Material) and analyzed by SDS- PAGE. (Fig. 1). The purified recombinant fusion protein was identified by western blot analysis using a monoclonal antibody against the 6 × His-Tag (Bioss, Beijing, China), and an expected band at 32.7 KDa could be observed (Additional file 1: Figure S6).

### Screening of positive hybridoma clones and purification of the mAb H8

Two hybridoma clones named B6 and H8 secreted monoclonal antibodies that recognized the recombinant fusion protein but not the fusion tags were obtained, but only mAb H8 could be used for the further applications.

**Fig. 2:**
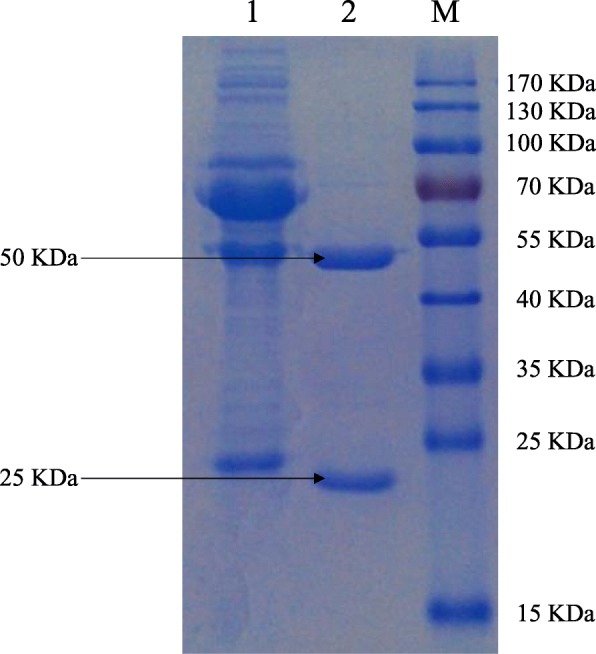
SDS-PAGE analysis of the purification of mAb H8. M, PageRuler™ Prestained Protein Ladder (Thermo Fisher Scientific); Lane 1, unpurified ascites fluid; Lane 2, purified ascites fluid. The positions of the target fragments are indicated by arrows

Hybridoma cell clones could be observed obviously after 4 d of fusion (Additional file 1: Figure S7), and the chromosome number of H8 cell line was about 82 ± 6, which was more than the number for mouse spleen cells or SP2/0 cells, and less than the total number for the two cells (Additional file 1: Figure S8). By injecting the hybridomas into paraffin primed BALB/c mice, ascetic fluid was produced. Monoclonal antibodies purified from this ascetic fluid (Fig. 2) were isotyped and mAb H8 was found to belong to the IgG1 subclass and had a kappa light chain. (Additional file 1: Figure S9). The results suggested that the hybridoma cell line H8 was successfully obtained.

### Analysis of mAb H8 by western blot

The western blot results demonstrated that two bands could be observed at 32.7 KDa and 17.2 KDa respectively (Additional file 1: Figure S10), which were in line with the expectations. This suggested the mAb H8 could react with both the prokaryotically expressed recombinant fusion protein cIL-17A and the eukaryotically expressed protein in supernatant of the cIL-17A-pEGFP-N1 transfected HEK293T cells.

### The applications of the mAb H8 in immunofluorescence and immunohistochemistry assays

**Fig. 3:**
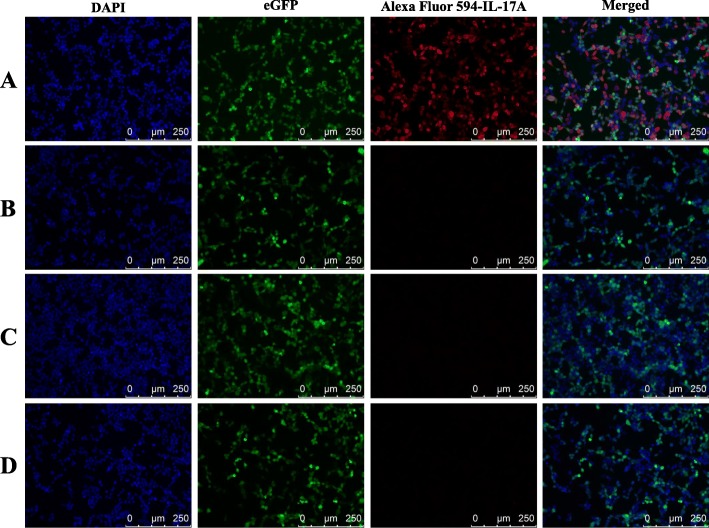
The results of immunofluorescence staining in HEK293T cells using mAb H8. **a** recombinant vector transfected HEK293T cells immunofluorescence staining by mAb H8; Lane 1, nuclei staining by DAPI; lane 2, the reporter protein eGFP; lane 3, IL-17A staining by mAb H8; lane 4, merged images (**b**) empty vector transfected HEK293T cells immunofluorescence staining by mAb H8 (**c**) empty vector transfected HEK293T cells immunofluorescence staining by isotype ascites fluid (**d**) recombinant vector transfected HEK293T cells immunofluorescence staining by isotype ascites fluid

**Fig. 4:**
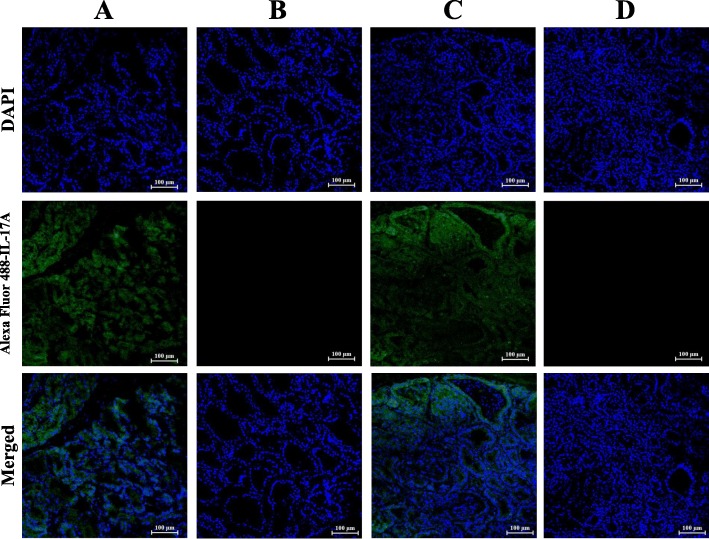
The results of immunofluorescence staining in mammary glands using mAb H8. **a** Non-infected gland immunofluorescence staining by mAb H8 and (**b**) isotype ascites fluid; (**c**) S. aureus-infected gland immunofluorescence staining by mAb H8 and (**d**) isotype ascites fluid; Lane 1, nuclei staining by DAPI; lane 2, IL-17A staining by mAb H8; lane 3, merged images

**Fig. 5:**
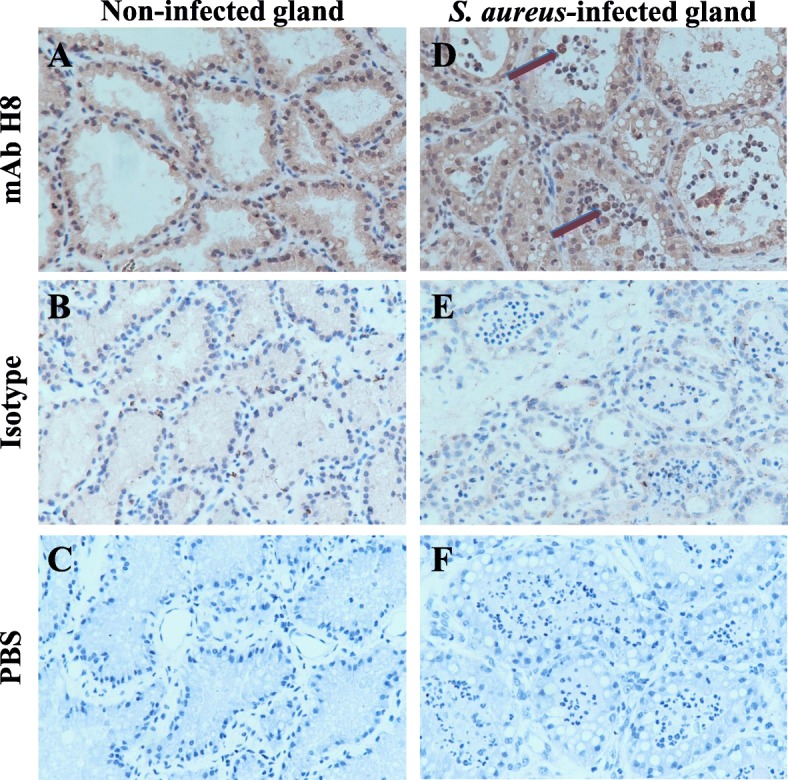
The results of immunohistochemistry staining in mammary glands using mAb H8. **a** Non-infected gland immunohistochemistry staining by mAb H8 and (**b**) isotype ascites fluid and (**c**) PBS; (**d**) *S. aureus*-infected gland immunohistochemistry staining by mAb H8 and (**e**) isotype ascites fluid and (**f**) PBS; Lane 1, Non-infected gland; lane 2, *S. aureus*-infected gland. The positions of the target cells are indicated by arrows (original magnification × 400)

Immunofluorescence and immunohistochemistry assays were performed to examine whether the mAb H8 could be used to recognize the natural cIL-17A protein and locate the IL-17A^+^ cells in situ. The results showed that mAb H8 could strongly recognize the eukaryotically expressed protein inside the cIL-17A-pEGFP-N1 transfected HEK293T cells (Fig. 3). We also analyzed the presence of IL-17A^+^ cells by immunofluorescence and immunohistochemistry assays and found that IL-17A^+^ cells were recruited into infected glands. In both control and infected glands, mammary epithelial cells were slightly IL-17A^+^ (Figs. 4 and 5), which were in line with the previous research [34].

The alveolar structure was integrated and few infiltrating cells could be observed in control glands, and rare cells could be stained as IL-17A^+^ cells. While in the infected gland, signs of tissue disorganization were apparent, characterized by the loss of alveolar structure in many areas and a lot of infiltrating cells, and some IL-17A^+^ cells were found in both connective area and inside the alveolar (Figs. 4 and 5).

## Discussion

There are limited commercial monoclonal antibodies available for the research of goat IL-17A so far. Although some researchers have tried to find the cross-reactivities of commercial human or mice monoclonal antibodies available for ruminants [35, 36], or used the commercial bovine IL-17A as an antigen to prepare mAbs that react with goat and sheep [37], there are only a few monoclonal antibodies available for caprine IL-17A. In this study, the prokaryotically expressed soluble recombinant fusion cIL-17A was used as an antigen to prepare monoclonal antibodies against goat IL-17A, and the monoclonal antibody H8 was successfully used in western blot, immunohistochemistry and immunofluorescence.

Prokaryotic expression has many advantages, including lower cost, a brief protocol, and ease of handling and purification compared with other methods, including eukaryotic expression. Although the CDS region of cIL-17A has been published before (GU269912.1), no studies have reported the isolation of cIL-17A protein, even as inclusion bodies. The efficiency of prokaryotic expression can be affected by various factors, including the features of the target gene, expression vector, host cell, inducing temperature, inducing concentration, and inducing time [38, 39]. In our system, the most commonly used host cell BL21 (DE3) was first chosen, but we found that no soluble protein was expressed, even when the inducing temperature was reduced to 16 °C (data not shown). Sequence analysis suggested that there were only 9 rare codons that would not theoretically influence protein expression, so we did not choose the Rosetta (DE3) host cell to replace BL21 (DE3). Another host cell, TransB (DE3), was chosen because it contained two reductase genes, thioredoxin reductase (trx B) and glutathione reductase (gor), which can form a strongly reductive conditions in the internal cell and be beneficial for the formation of disulfide bonds and soluble protein yield [40, 41]. Some researchers have obtained soluble expressed proteins in different areas by using the host cell TransB (DE3) [42–44].

In the present study, we found that the inducing temperature of the host cell TransB (DE3) should not be reduced to less than 16 °C, which was reflected by the fact that the bacteria grew very slowly at lower temperatures such as 14 °C or 12 °C (data not shown). The concentration of the inducer is also an important factor that influences soluble expression. In general, high concentrations of inducer would lead to the generation of inclusion bodies, while low concentrations of inducer would have no effects on induction [45, 46].

The prokaryotic expression vector PET 28a containing only 6 × His-tag was first chosen, but no soluble protein was obtained in either BL21 (DE3) or TransB (DE3) under any concentration of IPTG or at any inducing temperature. The vector PET 32a contains a tag protein trx.tag which is beneficial for the formation of disulfide bonds and facilitates soluble expression [47, 48]. Thus, we used this vector to replace PET 28a, and soluble recombinant fusion protein was obtained finally. However, the disadvantage of the tag is that because of the large 105 amino acid size, some epitopes and active sites may become sequestered. Although finally we have obtained an IL-17A-specific monoclonal antibody, there may still be some epitopes covered by the large fused tag protein. In addition, another problem is that there was no modification of the expressed protein, which is an “innate” defect of the prokaryotic expression and of course influenced the structure of the epitopes and protein activity.

We did not detect natural cIL-17A in blood and milk of dairy goats suffering from clinical mastitis caused by *Staphylococcus aureus* by using the mAb H8 for western blot analysis, maybe because of the low concentration of natural cIL-17A (data not shown).

Because the monoclonal antibody H8 can be used for western blot, IHC and IF analysis, it is speculated that mAb H8 may recognize a linear epitope located in the outer area of the protein, but we did not determine the sequence of the epitope recognized by mAb H8, which need further study.

In the experiment of immunohistochemistry analysis, the mammary epithelial cells also showed slightly IL-17A^+^, which corresponds to a research done on mouse mammary glands [34], but there are few study reports on the expression of IL-17 in mammary epithelial cells, so we could not provide a reasonably and perfect explanation so far.

A shortcoming of this study is that the specificity of the mAb H8 has not been fully evaluated. This is mainly resulted from the difficulty to obtain specific natural goat proteins, either purified or unpurified. However, as shown in Fig. 3, mAb H8 can only recognize eukaryotically expressed goat IL-17A and does not react with any unrelated proteins such as the reporter protein eGFP and other secreted proteins of HEK293T cells. As eukaryotically expressed proteins are very similar to natural proteins, we believe that the results of Fig. 3 could reflect a high specificity of mAb H8, although this needs more detailed evaluations in the future.

This study is the first to produce soluble recombinant fusion protein cIL-17A in *E. coli*, and the first to prepare a monoclonal antibody using caprine IL-17A. As mentioned earlier, the anti-cIL-17A monoclonal antibody H8 obtained by immunization of the recombinant fusion protein prepared in the present study could be an important tool to investigate the pathogenesis of a variety of IL-17A-associated diseases.

## Conclusions

In the present study, an optimized prokaryotic expression system was used to produce soluble recombinant fusion protein cIL-17A, which was used as an antigen to prepare monoclonal antibodies. And one strain of the monoclonal antibodies named H8 was successfully obtained and proved suitable for both IF and IHC.

## Methods

### Animals

Six female BALB/c mice were purchased from the laboratory animal center of the Air Force Medical University, XiAn city, Shaanxi Province, China. All mice were housed in a specific pathogen-free facility and treated in accordance with the guidelines of the Care and Use of Laboratory Animals of the Ministry of Health, China. All the mice mentioned in the present study were euthanized by the method of spine dislocation. Three healthy 3-year-old Guanzhong dairy goats in lactation were purchased from a farm nearby our university. After the study, the dairy goats used for establishment of clinical mastitis mentioned in this study were treated with antibiotic therapy until they were fully recovered.

### Main reagents

Lymphocyte separation medium (Ficoll-Hypaque Solution) was purchased from Shanghai HuaJing biological high-tech company (Shanghai, China). RNAiso Plus, PrimeSTAR® Max DNA Polymerase, Takara_Premix Taq™ (Takara Taq™ Version 2.0 plus dye), T_4_ DNA ligase, Reverse Transcriptase M-MLV (RNase H^−^), DL500 DNA Marker, DL15,000 DNA Marker, Premixed Protein Marker (Low) and the restriction enzymes EcoR I and Xho I were purchased from Takara Bio Inc. (Dalian, China). Concanavalin A (Con A), isopropyl-β-d-thiogalactoside (IPTG), imidazole, complete Freund’s adjuvant, incomplete Freund’s adjuvant, hypoxanthine aminopterin and thymidine (HAT) and Brefeldin A (BFA) were purchased from Sigma-Aldrich (St. Louis, Missouri, USA). PageRuler™ Prestained Protein Ladder was purchased from Thermo Fisher Scientific (Waltham, Massachusetts, USA). The DNA extraction kit was purchased from TIANGEN (Beijing, China). The Ni-NTA resin column was purchased from TransGen Biotech (Beijing, China). The monoclonal antibody against the 6 × His-Tag was purchased from Bioss (Beijing, China). Polyethylene glycol 1500 (PEG 1500) and IsoStrip™ Mouse Monoclonal Antibody Isotyping Kit were purchased from Roche (Basel, Switzerland). Goat anti-Mouse IgG Antibody HRP conjugate was purchased from Biosharp (ShenZhen, China). Alexa Fluor 594-conjugated Goat Anti-Mouse IgG (H + L) and Alexa Fluor 488-conjugated Goat Anti-Mouse IgG (H + L) were purchased from Proteintech (WuHan, China). The SP kit detection system was purchased from ZSGB-BIO (Beijing, China).

### Plasmids and cells

Recombinant *E. coli* DH5α containing the expression vector PET 32a was preserved in 8% glycerol at − 80 °C in our laboratory. *Staphylococcus aureus* (*S. aureus*) was isolated and characterized from a dairy goat suffering clinical mastitis and preserved in 8% glycerol at − 80 °C in our laboratory. Murine myeloma cell line SP2/0 and HEK293T cells were preserved in “10% DMSO + 90% Fetal Bovine Serum” at liquid nitrogen in our laboratory. Chemically competent *E. coli* TransB (DE3) cells were purchased from TransGen Biotech (Beijing, China).

### Preparation and characterization of monoclonal antibodies

The purified recombinant fusion protein cIL-17A was used as an antigen to produce monoclonal antibodies, and the procedures for preparation and purification of the recombinant fusion protein were detailed in Additional file 1. Six female 6 to 8-week-old BALB/c mice were injected at multiple sites subcutaneously and intraperitoneally with 100 μg of the purified recombinant fusion protein, thoroughly emulsified with an equal volume of complete Freund’s adjuvant (Sigma-Aldrich). Two boosts were given at days 14 and 28 with 100 μg of the purified recombinant fusion protein thoroughly emulsified with incomplete Freund’s adjuvant (Sigma-Aldrich). Three days after the last boost with 100 μg of the purified recombinant fusion protein, the splenocytes of the immunized BALB/c mice were fused with SP2/0 myeloma cells using polyethylene glycol 1500 (PEG 1500) (Roche). The hybridomas were selected in RPMI 1640 medium supplemented with hypoxanthine, aminopterin and thymidine (HAT) (Sigma-Aldrich). Positive clones were identified by indirect enzyme linked immunosorbent assay (iELISA) using the recombinant fusion protein and negative selected by the tag fusion proteins of PET 32a. After four subclonings, hybridomas producing mAbs were established and characterized, and chromosome analysis was identified.

Mice ascites fluid were generated by injecting the hybridomas into the paraffine primed BALB/c mice. After purification by the classical method of octanoic acid-ammonium sulfate precipitation, ascites fluid was used for mAb isotyping using an IsoStrip™ Mouse Monoclonal Antibody Isotyping Kit (Roche) according to the manufacturer’s instructions.

### Application of the monoclonal antibodies

Ascites fluid mentioned above was used as the source of murine monoclonal antibodies. By using Goat anti-Mouse IgG Antibody HRP conjugate (Biosharp) as the second antibody, the mAbs were used for western blot analysis by detecting the supernatant of cIL-17A-pEGFP-N1 transfected HEK293T cells. By using Alexa Fluor 594-conjugated Goat Anti-Mouse IgG (H + L, proteintech) and Alexa Fluor 488-conjugated Goat Anti-Mouse IgG (H + L, proteintech) as the secondary antibodies, the mAbs were used for immunofluorescence analysis by detecting the eukaryotically expressed protein inside the Brefeldin A (BFA, Sigma-Aldrich) treated cIL-17A-pEGFP-N1 transfected HEK293T cells and the natural cIL-17A located in mammary gland of dairy goat suffering *Staphylococcus aureus* caused clinical mastitis, respectively. By using SP kit detection system (ZSGB-BIO), the mAbs was used for immunohistochemistry analysis by detecting the natural cIL-17A located in mammary gland of dairy goat suffering from clinical mastitis caused by *Staphylococcus aureus*.

## Additional file

Additional file 1: **Methods.** Cloning of the CDS region of cIL-17A and construction of the expression plasmid. Expression of the recombinant fusion protein of cIL-17A. Optimization of the purification conditions. Purification and identification of the recombinant fusion protein of cIL-17A. **Results.** Construction of the expression plasmid. Optimization of the purification conditions. **Figure S1.** Cloning of the CDS region of caprine IL-17A. **Figure S2.** Colony PCR analysis of the recombinant *E. coli* cells. **Figure S3.** Double enzyme digestion analysis of the recombinant expression plasmid cIL-17-PET 32a. **Figure S4.** SDS-PAGE analysis of the expression of the recombinant fusion protein in *E. coli* TransB (DE3) induced at 16 °C for 42 h. **Figure S5.** Optimization of the purification conditions. **Figure S6.** Western blot analysis of the recombinant cIL-17A. **Figure S7.** The observation of hybridoma cell clones cultured in HAT medium (original magnification × 400). **Figure S8.** Chromosome analysis of the hybridoma cell line H8. **Figure S9.** Identification of the isotype of mAb H8. **Figure S10.** Western blot analysis of supernatants of HEK293T cells transfected with recombinant or empty vectors using mAb H8. (DOCX 24 kb)

## Data Availability

All data generated or analyzed during this study are included in this published article and its supplementary information files.

## Abbreviations

CDS: Coding sequence
cIL-17: Caprine interleukin-17
Con A: Concanavalin A
iELISA: Indirect enzyme linked immunosorbent assay
IF: Immunofluorescence
IHC: Immunohistochemistry
IL-17: Interleukin-17
ILCs: Innate lymphoid cells
IPTG: Isopropyl-β-d-thiogalactoside
LTi cells: Lymphoid tissue–inducer cells
mAb: Monoclonal antibody
NCR: Natural cytotoxicity receptor
NKT cells: Natural killer T cells
PBMCs: Peripheral blood mononuclear cells
PCR: Polymerase chain reaction
RORγt: RAR-related orphan receptor gamma t
SDS-PAGE: Sodium dodecyl sulfate-polyacrylamide gel electrophoresis
Tc17 cells: IL-17-secreting CD8^+^ cytotoxic T lymphocytes
Th17 cells: T helper 17 lymphocytes
γδ T cells: γδ TCR^+^ T lymphocytes
