# Supplementary material for: Preparation of a novel monoclonal antibody against caprine interleukin-17A and its applications in immunofluorescence and immunohistochemistry assays
Source: BMC Biotechnol. 2019 Jul 17;19:47. doi: 10.1186/s12896-019-0543-5 (PMC6637523; doi:10.1186/s12896-019-0543-5)
Supplement: Supplementary file 1 — Methods. Cloning of the CDS region of cIL-17A and construction of the expression plasmid. Expression of the recombinant fusion protein of cIL-17A. Optimization of the purification conditions. Purification and identification of the recombinant fusion protein of cIL-17A. Results. Construction of the expression plasmid. Optimization of the purification conditions. Figure S1. Cloning of the CDS region of caprine IL-17A. Figure S2. Colony PCR analysis of the recombinant E. coli cells. Figure S3. Double enzyme digestion analysis of the recombinant expression plasmid cIL-17-PET 32a. Figure S4. SDS-PAGE analysis of the expression of the recombinant fusion protein in E. coli TransB (DE3) induced at 16 °C for 42 h. Figure S5. Optimization of the purification conditions. Figure S6. Western blot analysis of the recombinant cIL-17A. Figure S7. The observation of hybridoma cell clones cultured in HAT medium (original magnification × 400). Figure S8. Chromosome analysis of the hybridoma cell line H8. Figure S9. Identification of the isotype of mAb H8. Figure S10. Western blot analysis of supernatants of HEK293T cells transfected with recombinant or empty vectors using mAb H8. (DOCX 24 kb) [file 12896_2019_543_MOESM1_ESM.docx]

**Additional file 1**

**Preparation of a novel monoclonal antibody against caprine interleukin-17A and its applications in immunofluorescence and immunohistochemistry assays**

Yang Gao ^1^, FengFeng Sang ^1^, DeLan Meng ^1^, Yi Wang ^1^, WenTao Ma ^1*^, DeKun Chen ^1*^,

^1^ *College of Veterinary Medicine,* *Northwest A&F University, Yangling, Shaanxi Province, 712100, People’s Republic of China*

**Methods**

**Main reagents**

Lymphocyte separation medium ([Ficoll-Hypaque Solution](javascript:;)) was purchased from Shanghai HuaJing biological high-tech company (Shanghai, China). RNAiso Plus, PrimeSTAR^®^ Max DNA Polymerase, Takara_Premix Taq™ (Takara Taq™ Version 2.0 plus dye), T_4_ DNA ligase, Reverse Transcriptase M-MLV (RNase H^-^), DL500 DNA Marker, DL15,000 DNA Marker, Premixed Protein Marker (Low) and the restriction enzymes EcoR I and Xho I were purchased from Takara Bio Inc. (Dalian, China). Concanavalin A (Con A), isopropyl-β-d-thiogalactoside (IPTG), imidazole, complete Freund’s adjuvant, incomplete Freund’s adjuvant, hypoxanthine aminopterin and thymidine (HAT) and Brefeldin A (BFA) were purchased from Sigma-Aldrich (St. Louis, Missouri, USA). PageRuler™ Prestained Protein Ladder was purchased from Thermo Fisher Scientific (Waltham, Massachusetts, USA). The DNA extraction kit was purchased from TIANGEN (Beijing, China). The Ni-NTA resin column was purchased from TransGen Biotech (Beijing, China). The monoclonal antibody against the 6×His-Tag was purchased from Bioss (Beijing, China). Polyethylene glycol 1500 (PEG 1500) and IsoStrip™ Mouse Monoclonal Antibody Isotyping Kit were purchased from Roche (Basel, Switzerland). Goat anti-Mouse IgG Antibody HRP conjugate was purchased from Biosharp (ShenZhen, China). Alexa Fluor 594-conjugated Goat Anti-Mouse IgG (H+L) and Alexa Fluor 488-conjugated Goat Anti-Mouse IgG (H+L) were purchased from Proteintech (WuHan, China). The SP kit detection system was purchased from ZSGB-BIO (Beijing, China).

**Cloning of the** **CDS region of cIL-17A and construction of the expression plasmid**

A pair of primers were designed according to the published sequence (signal peptide sequence removed) from GenBank (GU269912.1): forward primer: (5’-CCG*GAATTC*GGGGTCATCATCCCACAGAGTC-3’) and reverse primer: (5’-CCG*CTCGAG*TTAAGCCACATGGCGGACAAT-3’). Peripheral blood mononuclear cells (PBMCs) from dairy goats were separated from heparinized blood and stimulated with Con A at a concentration of 10 μg/ml for 24 h. Then, total RNA was extracted by the Trizol method, and cDNA was synthesized by using Reverse Transcriptase M-MLV (RNase H^-^). cDNA was used as the template to amplify the CDS region of cIL-17A. The PCR program was as follows: 94 °C for 5 min, followed by 32 cycles of 94 °C for 30 s, 62 °C for 30 s, 72 °C for 30 s, and finally 10 min at 72 °C and hold at 16 °C. The PCR products were analyzed by electrophoresis on a 2% agarose gel and purified using a DNA extraction kit. The purified PCR products and the prokaryotic expression vector PET 32a preserved in our laboratory were double digested at 37 °C for 12 h and 2 h, respectively, and then analyzed by electrophoresis and purified using a DNA extraction kit and catalyzed by T_4_ DNA ligase at 4 °C for 12 h. Then, the connected products were transformed into the chemically competent *E. coli* TransB (DE3) cells. A total of 20 transformants were picked and analyzed by colony PCR and double enzyme digestion. The identified positives were sequenced by GenScript (Nanjing, China Co., Ltd).

**Expression of the recombinant fusion protein of cIL-17A**

### A single colony of the sequenced recombinant strain from a Luria Bertani (LB) agar plate (10 g/l NaCl, 5 g/l yeast extract, 10 g/l tryptone, and 10 g/l agar powder, pH 7.4) containing 50 mg/ml ampicillin and kanamycin was picked and inoculated in 2 ml of LB liquid medium (10 g/l NaCl, 5 g/l yeast extract, and 10 g/l tryptone, pH 7.4) containing 50 mg/ml ampicillin and kanamycin at 37 °C under shaking (220 rpm) overnight. We inoculated 5 ml of fresh LB medium with 50 μl of the overnight culture and shook the mixture until the cell density at 600 nm (OD_600_) reached 0.5-1.0. Then, IPTG at a final concentration of 1.0 mmol/L was added to induce the expression of the recombinant fusion protein at 37 °C for 6 h. The culture was harvested and washed three times and resuspended with 500 μl of PBS. After sonication, the mixture was centrifuged at 12,000 g at 4 °C for 30 min, and then, the supernatant and pellet were both analyzed by SDS-PAGE. A series of temperatures, inducing times and IPTG concentrations were chosen to optimize the expression conditions.

**Optimization of the purification conditions**

A larger [cultivation](file:///D:\Program%20Files\%E6%9C%89%E9%81%93%E8%AF%8D%E5%85%B8\Dict\7.1.0.0421\resultui\dict\?keyword=cultivation) of 20 ml of induced cIL-17-PET 32a-TransB (DE3) with IPTG at a concentration of 0.3 mmol/L at 16 °C for 42 h was sonicated, and then, the soluble protein in the supernatant was obtained by centrifugation. A series of imidazole concentrations of 20, 40, 60, 80, 200, 400, 500 and 1000 mmol/L were chosen to optimize the purification conditions.

**Purification and identification of the recombinant fusion protein of cIL-17A**

The recombinant fusion protein was expressed at a large-scale according to the optimized conditions and purified by affinity chromatography with a Ni-NTA resin column according to the manufacturer’s instructions (TransGen Biotech, Beijing, China). Then, the purified recombinant fusion protein was analyzed by western blot using a monoclonal antibody against the 6×His-Tag (Bioss, Beijing, China).

**Results**

**Construction of the expression plasmid**

A DNA fragment at the expected size of 393 bp was obtained by PCR and then analyzed by 2% agarose electrophoresis (Fig. S1). The fragments of interest were inserted into the prokaryotic expression vector PET 32a and then transformed into *E. coli* TransB (DE3). The recombinant plasmids containing the CDS region (without the signal sequence) of cIL-17A were confirmed by colony PCR (Fig. S2) and restriction enzyme analysis with EcoR I and Xho I (Fig. S3). Then, the colonies identified as positive by both colony PCR and restriction enzyme analysis were further confirmed by DNA sequencing. The identified recombinant expression plasmid was named “cIL-17-PET 32a”.

**Optimization of the purification conditions**

We found that most of the unrelated proteins could be washed away with only nominal loss of target protein when the imidazole solution at a concentration of 80 mmol/L was flowed through the column. On the other hand, all of the target protein could be eluted by the imidazole solution at a concentration of 500 mmol/L (Fig. S5). Thus, the imidazole solution at a concentration of 80 mmol/L was selected to wash away the unrelated proteins while a concentration of 500 mmol/L was used to elute the target protein.

Figure S1. Cloning of the CDS region of caprine IL-17A.

M, DL500 DNA marker; N, Negative control; Lane 1, the CDS region of caprine IL-17A without the signal sequence. The position of the target fragment is indicated by an arrow.

Figure S2. Colony PCR analysis of the recombinant *E. coli* cells.

Lane 1-5, five different monoclonal colonies; N, Negative controls; M, DL500 DNA marker. The position of the target fragment is indicated by an arrow.

Figure S3. [Double enzyme digestion](javascript:;) analysis of the recombinant expression plasmid cIL-17-PET 32a.

M, DL15,000 DNA marker; Lane 1, undigested recombinant plasmids; Lane 2, digested recombinant plasmids with the [restriction enzyme](javascript:;)s EcoR I and Xho I. The positions of the target fragments are indicated by arrows.

Figure S4. SDS-PAGE analysis of the expression of the recombinant fusion protein in *E. coli* TransB (DE3) incued at 16 °C for 42 h.

M, Premixed protein marker (low); Lane 1, the uninduced cIL-17-PET 32a-TransB (DE3); Lane 2, induced PET 32a-TransB (DE3) with IPTG at a concentration of 0.5 mmol/L; Lane 3-6, the pellet of induced cIL-17-PET 32a-TransB (DE3) with IPTG at different concentrations of 0.01, 0.05, 0.3 and 0.7 mmol/L, respectively; Lane 8-11, the supernatants of induced cIL-17-PET 32a-TransB (DE3) with IPTG at different concentrations of 0.01, 0.05, 0.3 and 0.7 mmol/L, respectively. The position of the recombinant fusion protein is indicated by an arrow.

Figure S5. Optimization of the purification conditions.

M, Premixed protein marker (low); Lane 1, the uninduced cIL-17-PET 32a-TransB (DE3); Lane 2, induced PET 32a-TransB (DE3) with IPTG at a concentration of 0.5 mmol/L at 16 °C for 42 h; Lane 3, unpurified recombinant cIL-17; Lane 4, eluents for the unpurified supernatant flowed through the column; Lane 5-13, eluents after the column was washed with imidazole at different concentrations of 20, 40, 60, 80, 200, 400, 500 and 1000 mmol/L, respectively. The position of the target fragment is indicated by an arrow.

Figure S6. Western blot analysis of the recombinant cIL-17A.

Lane 1, fusion tags of PET 32a; Lane 2, inclusion bodies of recombinant cIL-17A; Lane 3, soluble recombinant cIL-17A; Lane 4, BSA as a negative control. The position of the target fragment is indicated by an arrow.

Figure S7. The observation of hybridoma cell clones cultured in HAT medium (original magnification ×400).

Figure S8. Chromosome analysis of the hybridoma cell line H8.

Figure S9. Identification of the isotype of mAb H8.

Figure S10. Western blot analysis of supernatants of HEK293T cells transfected with recombinant or empty vectors using mAb H8.

Lane 1, purified recombinant fusion cIL-17A as positive control; lane 2 and 3, supernatants of HEK293T cells transfected with empty vectors; lane 4, 5 and 6, supernatants of HEK293T cells transfected with recombinant vectors. The positions of the target fragments are indicated by arrows.
